# Normative Values and Test/Retest Reliability of a New Test: Spatial Processing of Sentences in Noise in Portuguese

**DOI:** 10.1155/bmri/7880012

**Published:** 2025-06-28

**Authors:** Leticia Reis Borges-Ifanger, Bruno Sanches Masiero, Maria Francisca Colella-Santos

**Affiliations:** ^1^Faculty of Hearing Sciences, Pontifical Catholic University of Campinas, Campinas, São Paulo, Brazil; ^2^Communication Acoustics Lab, Universidade Estadual de Campinas, Campinas, São Paulo, Brazil; ^3^School of Medical Sciences, Universidade Estadual de Campinas, Campinas, São Paulo, Brazil

**Keywords:** children, spatial processing

## Abstract

**Objective:** This article establishes normative values for children and analyzes test–retest results for a new test, the Spatial Processing of Sentences in Noise in Portuguese (PROSER).

**Methods:** To establish normative criteria, we evaluated 66 Brazilian Portuguese-speaking children aged 7–10 years using audiological assessments, school performance tests, and the PROSER test. A subset of 22 children participated in a test–retest evaluation.

**Results:** Examining all 66 participants, we found significant differences in the speech reception threshold (SRT) means between the 0° and ±90° interference conditions when analyzing all four test conditions. Considering the age group, the performance of 10-year-old children was superior to that of both 7- and 8-year-old children. Test–retest comparison showed slight improvements (0.11–1.13 dB) in the retest phase across most conditions and advantage measures. The difference between the average SRT in the test and retest was statistically significant only in Condition 3-DV0°.

**Conclusions:** The study established normative values for children aged 7–10 years, confirming PROSER as a procedure with adequate test–retest reliability.

## 1. Introduction

Central auditory processing disorder (CAPD) is a dysfunction of the central auditory system that causes hearing difficulties [1]. Children and adults with this disorder often struggle to comprehend speech in noisy environments [2], which can be a functional manifestation of spatial processing disorder (SPD). Individuals with SPD struggle to focus on primary messages while suppressing competing sounds from different locations [3]. SPD occurs because brain structures cannot effectively compare stimulus arrival times between ears or properly utilize spatial relation cues [3–5], reducing the ability to separate target speech from background noise.

SPD affects people with normal hearing thresholds [4, 6], those with mild to moderately severe hearing loss [5], and individuals with various clinical comorbidities [7, 8]. Research has identified numerous children with hearing complaints and SPD [4, 9], with recurrent otitis media in early childhood as a frequent etiology for SPD [10, 11].

The Listening in Spatialized Noise-Sentences Test (LISN-S) [3, 6, 12] was developed in Australia with the primary purpose of evaluating the speech in noise abilities, including the spatial processing abilities of individuals with complaints related to CAPD. It is a speech-in-noise test, applied via dedicated computer software and a headset, producing a three-dimensional virtual auditory environment. A simple repetition response protocol is used to obtain the speech reception threshold (SRT) and find the signal-to-noise (S/N) ratio that delivers 50% intelligibility for sentences with competitive speech stimuli (children's stories).

A test–retest reliability study of the LISN-S revealed that it is a suitable test for monitoring spatial processing skills over time, in addition to determining whether the change in outcome was due to remediation or the variation that may occur in outcomes due to the effect of practice [12].

The LISN-S is currently available only in English [3, 12, 13], with no equivalent assessment tool in Brazilian Portuguese (PT-BR). To address this gap, we partnered with one of the LISN-S authors to develop the test for spatial processing of sentences in noise (PROSER) in PT-BR. The development process involved three main phases: (1) creating specialized assessment software, (2) developing 188 PT-BR sentences and selecting two children's stories as competitive stimuli, and (3) validating the relative intelligibility between sentences to ensure consistent difficulty levels [14]. This PT-BR adaptation will facilitate identifying spatial listening deficits in the Brazilian population, enabling more comprehensive and effective intervention planning and rehabilitation.

This study is aimed at establishing normative values for children using PROSER across its four spatial listening conditions and the derived spatial, talker, and total advantage measures. We analyze these values by considering age and gender factors while also examining test–retest reliability to verify the stability of measurements over time.

## 2. Method

### 2.1. Ethics Statement

The Research Ethics Committee of Unicamp approved this prospective cross-sectional study under Number 3.462.572. Written parents'/guardians' consent was obtained for all participants included in this study under the age of 16.

### 2.2. Participants

Sixty-six students (aged 7–10 years, both male and female) were recruited from Sergio Porto State Primary School between October 2019 and November 2021.

The inclusion criteria were as follows:

− age between 7 and 10 years;
− PT-BR as a first language;
− hearing auditory thresholds within normal ranges [15];
− normal middle ear function confirmed by Type A tympanogram (peak compliance: 0.3–1.3 m℧, pressure: −100 to +50 daPa) with present ipsi- and contralateral acoustic reflexes at 70–100 dB above auditory threshold [16];
− present otoacoustic emissions, with response amplitude (signal/noise ratio) equal to or greater than 6 dB sound pressure level (SPL) in three frequency bands and probe stability equal to or greater than 70% [17];
− typical automatic auditory brainstem response (AABR) on the day of the assessment;
− no history of middle ear disease and/or attention deficit.

School performance was assessed using the School Performance Test, including reading and writing tasks. Only children demonstrating average or superior performance were included in the study [18]. We excluded children with behavioral or neurological disorders, genetic syndromes, those using psychoactive medications, and those in speech therapy who failed to meet other inclusion criteria.

### 2.3. Procedures and Measures

#### 2.3.1. Initial Procedures

Audiological assessments were conducted at the audiology laboratories of the Department of Human Development and Rehabilitation, School of Medical Sciences, Unicamp. The PROSER and school performance testing took place in a quiet room at the school.

Equipment used for the prescreening included an Interacoustics AC40 Audiometer with TDH 39P headphones for pure-tone audiometry and a tympanometer to evaluate middle ear function. The TITAN device (also Interacoustics) was used for otoacoustic emissions and AABR testing. All equipment was calibrated according to ISO-389 and IEC-645 standards.

#### 2.3.2. PROSER Test

The test comprised 120 phrases (7 three-word, 31 four-word, 51 five-word, 29 six-word, and 2 seven-word phrases) developed by a speech therapist and recorded by a single female speaker. Two children's stories, whose titles can be freely translated to “The King's New Clothes” and “The Rooster and the Fox” [19], served as competitive messages and were recorded by three female speakers, including the one who recorded the target sentences. Both the target sentences and competitive messages were presented simultaneously to both ears.

By using suitable head-related transfer functions (HRTFs), the target sentences were processed in such a way as to be perceived as always coming from directly in front of the listener (0° azimuth). In contrast, the children's stories were processed to be perceived as coming simultaneously from 0° azimuth or ±90° azimuth. The competitive message also varied in terms of the vocal identity of the speaker, as it was presented with the same voice (SV) as the target sentence and different voices (DVs). In this way, there was a variation in the presentation of the competitive speech concerning the target sentence, considering the spatial location and the vocal identity of the speaker, which resulted in four listening conditions:

Condition 1—SV0°or SRT low cue: SV for the target sentence and the competitive message. The competitive message seems to come from the 0° azimuth position. In this condition, no spatial or vocal cues are provided.

Condition 2—SV±90°: SV for the target sentence and the competitive message. The competitive message seems to come from opposite sides (position ±90°). In this condition, the spatial cue is provided.

Condition 3—DV0°: The voice in the competitive message differs from that in the target sentence. The competitive message seems to come at 0° azimuth. In this condition, only the speaker cue is provided.

Condition 4—DV±90° or SRT many clues: The voice in the competitive message differs from that in the target sentence. The competitive message seems to come from opposite sides (position ±90°). In this condition, both speaker and spatial cues are provided.

Before beginning the test, participants were informed they would hear target sentences presented simultaneously with competitive stories, preceded by a warning signal. They were instructed to listen to each complete sentence before repeating it as accurately as possible. The assessment began with a training phase consisting of three practice sentences presented at a fixed S/N ratio of +7 dB, with competitive stories delivered at 65 dB SPL and target sentences at 72 dB SPL.

After the training phase, adaptive testing began with the fourth sentence. Initially, the level of target sentences decreased by 4 dB until the first reversal occurred (when less than 50% of words were correctly repeated). Thereafter, 2 dB steps were implemented following an adaptive procedure: when fewer than 50% of words were correctly identified, the intensity of the next target stimulus increased by 2 dB; when more than 50% of words were correctly identified, the intensity decreased by 2 dB; and when exactly 50% of words were correctly identified, the intensity remained unchanged.

The SRT was defined as the S/N ratio that produced 50% intelligibility and was calculated as the average of at least three intermediate points. In turn, each intermediate point was calculated as the average of the level of a positive reversal (changes the gain from negative to positive) and the level of the subsequent negative reversal (changes the gain from positive to negative).

Following each response, the evaluator entered the number of correctly repeated words into the software. Testing in each condition concluded when either 30 sentences were completed or the participant finished the training phase, plus a minimum of 17 additional sentences with a standard error (calculated automatically in real-time) of less than 1 dB.

This procedure was applied across all four test conditions, with presentation order counterbalanced among participants. PROSER was administered using Sennheiser HD 280 PRO headphones connected to a personal computer via an RME MADIface Pro audio interface.

**Figure 1:**
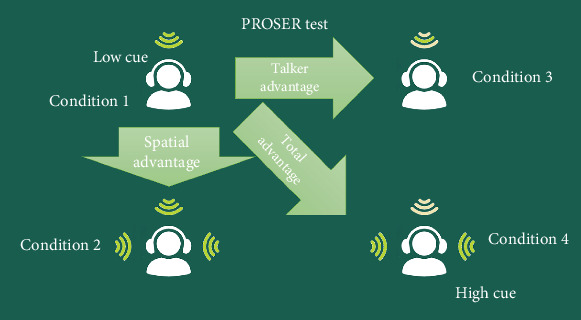
The four presentation conditions of target sentences and children's stories and three measures of advantage calculated from them.

Performance was evaluated using the SRT measured in all four test conditions and three derived advantage measures: spatial, speaker, and total (Figure 1). Spatial advantage was calculated as the difference in SRT (in decibels) between Conditions 1 and 2 (SV0°–SV±90°). Speaker advantage was determined by the SRT difference between Conditions 1 and 3 (SV0°–DV0°). Total advantage represented the difference between Conditions 1 and 4 (SV0°–DV±90°). For test–retest reliability analysis, 22 children were reassessed using identical procedures 2–3 months after their initial evaluation.

Presentation form of phrases and competitive message for each PROSER condition is presented in Table 1. For all four conditions, target sentences are always emitted by the same female speaker at 0° azimuth.

### 2.4. Statistical Analyses

The sample was described using frequency tables for categorical variables and descriptive measures for numerical variables. Group comparisons based on age, sex, and application order were conducted using Mann–Whitney or Kruskal–Wallis tests, with Dunn's test and Bonferroni's correction applied when necessary.

For test–retest analysis, we calculated descriptive measures for test values, retest values, and the differences between them across the sample. Comparisons between test and retest for each condition were analyzed using Wilcoxon's paired tests. We also generated scatterplots with 95% confidence intervals. All analyses used a significance level of 5% (significant results highlighted in bold in tables), performed using SAS System Version 9.4 and R Version 4.2.0.

## 3. Results

### 3.1. Part I: Results Relating to PROSER Normality Data

The PROSER test was applied to 66 children aged 7–10 years, 35 males and 31 females, whose distribution is presented in Table 2.

Table 3 presents descriptive statistics for all age groups combined, including mean SRT, standard deviation (SD), minimum, median, and maximum values (all in decibels), along with the for each of the four PROSER conditions: DV0°, DV±90°, SV±90°, and SV0°.  Table 4 analyzes each condition combination using the Wilcoxon test to compare competitive stimulus locations (0° vs. ±90°) and competitive voice characteristics (SV vs. DVs in target stimulus).

Table 3 shows that the ±90° condition yielded lower SRT values than the 0° condition in both SV and DV configurations, indicating that speech understanding in noise is easier when target speech and competitive noise originate from different spatial locations (±90°) compared to the same location (0°).  Table 4 reveals statistically significant differences only between spatial conditions (0° vs. ±90°), with no significant differences observed between speaker voice conditions.

Table 5 compares performance across age groups, demonstrating that 10-year-old children performed significantly better than younger groups in Conditions 1—SV0°, 2—SV±90°, and 3—DV0°, with lower SRT values indicating that age influences spatial processing abilities.

Table 6 presents results regarding the order of presentation for PROSER test conditions. Age ranges were combined to ensure adequate sample sizes in each condition. To evaluate whether practice effects influenced performance, we compared mean SRT values across all four conditions (SV0°, SV±90°, DV0°, and DV±90°) based on their testing sequence (first, second, third, or fourth position) using the Kruskal–Wallis test. The *p* values indicate that presentation order did not significantly affect mean SRT results.

### 3.2. Part II: Test/Retest

The retest was completed by 22 of the 66 children who participated in the normality study of the PROSER test. This subgroup included five 7-year-olds, five 8-year-olds, six 9-year-olds, and six 10-year-olds, with a mean age of 8.5 years.

Table 7 presents the results from both test and retest phases using the Wilcoxon test. Age ranges were combined to ensure adequate sample sizes in each condition. Comparing test and retest performance, we observed small mean differences ranging from 0.11 to 1.13 dB, with children generally performing better during the retest across all conditions and advantage measures. Statistical significance was reached only for Condition 3—DV0°, with no significant differences observed in the other conditions.

## 4. Discussion

The PROSER test was administered to 66 PT-BR-speaking children, aged 7–10 years, with normal hearing thresholds and middle ear conditions, normal auditory pathways to the brainstem, and satisfactory academic performance. This testing established normative values that will serve as reference standards when evaluating children with CAPD-related complaints, particularly those struggling to understand speech in noisy environments.

Including the PROSER test in the battery of CAP assessment in children will make it possible to know the diagnosis of SPD and thus promote a more complete diagnosis that will enable more assertive therapeutic planning while providing appropriate guidance for each case. Research indicates that approximately 17% of children undergoing CAPD evaluation demonstrate SPD, with many having a history of otitis media [15].

Our results demonstrate that the spatial location between target stimuli (sentences) and competitive stimuli (stories) significantly influenced performance. Children performed better when target stimuli were presented at the ±90° position, regardless of whether the same or different speakers delivered the competitive stimuli.

No similar effect was observed for speaker vocal identity (SV vs. DVs in target stimulus). Children performed similarly in both same-speaker and different-speaker conditions, regardless of whether stimuli were presented at 0° or ±90° positions.

Our results differ from findings in the Australian and American LISN-S versions, where both spatial location and speaker identity influenced performance in children aged 6–10 years [3, 13]. In our PROSER test, the female voice actresses who recorded target stimuli and competitive stories had similar vocal characteristics in terms of pitch and timing. Since conditions and advantages involving spatial location are fundamental for spatial processing assessment, while speaker identity serves primarily as a response-facilitating cue, we understand that this difference will not compromise PROSER's effectiveness when evaluating PT-BR-speaking children.

Furthermore, the talker advantage condition is also not considered in the latest version of LISN-S [20], the Listening in Spatialized Noise-Universal Test (LISN-U). This updated version eliminates the DV conditions, presenting target and competitive stimuli using only the SV. Following this development, a potential modification for PROSER would be to simplify testing by utilizing only the SV0° and SV±90° conditions and focusing exclusively on spatial advantage measurements.

In the age variable analyses, it was observed in PROSER that the performance of children aged 10 years was better in the studied Conditions 1—SV0°, 2—SV±90°, and 3—DV0°. The largest difference in SRT between ages was 2.57 dB in the SV±90° condition and 1.97 dB in the SV0° condition. Cameron and Dillon [3] applied the Australian version of LISN-S to children aged 5–11 years. They verified that 5-year-olds had a statistically significant difference in performance in all conditions and measures of advantage, except total advantage, when compared with virtually all other age groups studied.

The American version of LISN-S [13], which studied children aged 6–11 years, found that in Conditions 1—SV0° and 4—DV±90° and speaker and spatial advantage, age significantly affected children's performance, involving mainly 6-year-olds. In this study, the age group studied did not include children aged 5 and 6 years, which may explain the similar performance between the age groups in the PROSER test. Our study's narrower age range (7–10 years) may explain the more similar performance across age groups in the PROSER test. Children in this age range likely have comparable development of binaural interaction mechanisms, enabling them to perceive and compare subtle differences in stimulus arrival time and intensity between ears, facilitating auditory stimulus localization in noisy environments.

Analysis of presentation order revealed no significant effect on children's performance across PROSER conditions. Nevertheless, to enhance task comprehension, particularly for children who struggle with utilizing spatial cues to identify target stimuli, we recommend presenting conditions in order of increasing difficulty: (first) DV±90°, (second) SV±90°, (third) DV0°, and (fourth) SV0° [3].

Mean and SD values are commonly used to define the normality criteria. Still, no research justifies using one SD, one and a half, or two. In this first study, the authors opted to consider only the results obtained in the total sample due to the reduced differences in the analysis of the gender and age group variables (results differed from the calculated values mean SPD, mainly in Conditions 1 and 4 and in spatial and total advantage measures) [3]. New studies with children with typical development, hearing complaints, and other clinical entities will help us verify whether gender and age range variables should be considered in the normality criterion.

Comparing children's performance between test and retest phases revealed similar results with no statistical differences in most conditions, except for Condition 3—DV0°. The consistency of results across multiple evaluations demonstrates the homogeneity of PROSER test measurements and confirms its adequate reliability. The average differences between test and retest results, along with SD values, can serve as reference points to determine whether improvements in retest performance stem from test/retest effects or from developmental changes, auditory skill stimulation, or compensatory strategy implementation. A test with high test/retest reliability can be used more confidently across various studies, populations, and contexts, enhancing research replicability.

The mean SRT differences observed in our study (ranging from 0.11 to 1.13 dB) in Table 8 align with those found in the Australian and American LISN-S versions, which reported minor differences ranging from 0.1 to 1.1 dB and 0.1 to 0.7 dB, respectively. In the Australian version, the difference in mean SRT/advantage was statistically significant for all measures except spatial advantage [11], while the American version showed no statistically significant differences in mean SRT across any performance measures [13]. The difference between the results in the Australian and American versions was not statistically significant, considering age.

This initial PROSER study establishes normative criteria for classifying spatial processing as normal or altered. Future research should include larger samples of children aged 6–10 years to determine whether differentiated normative criteria are necessary across age groups. Projects evaluating children with CAPD, otitis media, and academic difficulties are underway to establish test sensitivity and specificity data. Additionally, we are developing a project focused on creating effective strategies for spatial processing stimulation and remediation.

## 5. Conclusion

Based on the analysis of the results obtained, we established normative criteria for children aged 7–10 years and verified that PROSER is a procedure with adequate test/retest reliability.

## Figures and Tables

**Table 1:** Form of phrases and competitive message for each PROSER condition.

**PROSER conditions**	**Stories—Female voice**	**Stories—Position**	**Stories**
Condition 1—SV0°	1	0°	“The King's New Clothes”
	1	0°	“Rooster and the Fox”

Condition 2—SV±90°	1	−90°	“The King's New Clothes”
	1	+90°	“Rooster and the Fox”

Condition 3—DV0°	2	0°	“The King's New Clothes”
	3	0°	“Rooster and the Fox”

Condition 4—DV±90°	2	−90°	“The King's New Clothes”
	3	+90°	“Rooster and the Fox”

**Table 2:** Characterization of the sample, considering male and female sex and age range.

**Sex/age (years)**	**7**	**8**	**9**	**10**	**Total**
Male	14	14	5	2	35
Female	14	5	5	7	31
Total	28	19	10	9	66

*Note:* Mean and median: 8.5 years.

**Table 3:** Descriptive measures of SRT, in decibels, and average number of sentences presented (excluding the training phase) for the 66 children in each of the four conditions of the PROSER test and measures of advantage.

**Condition**	**N**	**Min.**	**Q1**	**Mean**	**Median**	**Q3**	**Max**	**SD**
Condition 1—SV0°	66	−4.68	−2.89	−1.98	−1.78	−1.28	1.53	1.45
Condition 2—SV±90°	66	−18.43	−13.76	−11.96	−12.38	−10.25	−5.23	2.72
Condition 3—DV0°	66	−5.50	−2.67	−1.70	−1.69	−0.67	1.89	1.48
Condition 4—DV±90°	66	−16.74	−13.41	−12.04	−12.18	−10.53	−5.11	2.37
Spatial advantage	66	4.36	8.43	9.98	10.10	11.22	16.66	2.34
Talker advantage	66	−2.68	−1.09	−0.28	−0.34	0.53	2.17	1.14
Total advantage	66	4.11	8.56	10.06	10.06	11.32	14.66	2.09

Abbreviations: Max, maximum; Min, minimum; *N*, number of subjects; Q1, first quarter; Q3, third quarter; SD, standard deviation.

**Table 4:** Descriptive analysis of each combination of conditions comparing competitive stimulus location (0° vs. ±90°) and voice of competitive stimulus (same vs. different voices from the target stimulus).

**Combination**	**V** ** value**	**p** ** value**
0° × 90°	8778.0	< 0.001
0° × 90° (only SV)	2211.0	< 0.001
0° × 90° (only DV)	2211.0	< 0.001
SV × DV	4669.5	0.43
SV × DV (only 0°)	816.0	0.06
SV × DV (only 90°)	1140.0	0.66

*Note:* Wilcoxon's test.

Abbreviations: DVs, different voices; SV, same voice.

**Table 5:** Descriptive measures of the SRT in the test conditions and advantage measures, considering the age group.

**Condition**	**7**	**8**	**9**	**10**	**(** **p** ** value)** ^**a**^
	**Mean (SD)**	**Mean (SD)**	**Mean (SD)**	**Mean (SD)**	
Condition 1—SV0°	−1.38 (1.41)	−1.74 (1.26)	−2.87 (1.09)	−3.35 (0.98)	10 × 7 (*p* = 0.0009)^b^ 10 × 8 (*p* = 0.0073)^b^ 9 × 7 (*p* = 0.0082)^b^

Condition 2—SV±90°	−11.49 (3)	−11.07 (2.72)	−13.05 (1.4)	−14.06 (1.33)	10 × 7 (*p* = 0.0153)^b^ 10 × 8 (*p* = 0.0095)^b^

Condition 3—DV0°	−1.13 (1.54)	−1.83 (1.53)	−1.91 (1.06)	−2.96 (0.36)	10 × 7 (*p* = 0.0008)^b^

Condition 4—DV±90°	−11.54 (2.36)	−11.45 (2.6)	−13.23 (1.12)	−13.51 (2.03)	0.5645

Spatial advantage	10.11 (2.55)	9.33 (2.7)	10.19 (1.31)	10.71 (1.57)	0.9929

Talker advantage	−0.25 (0.97)	0.09 (1.3)	−0.96 (1.14)	−0.39 (1.12)	0.1017

Total advantage	10.16 (2.02)	9.71 (2.55)	10.37 (1.02)	10.17 (2.33)	0.4610

Abbreviation: SD, standard deviation.

^a^Kruskal–Wallis test.

^b^Post hoc test.

**Table 6:** SRT under the test conditions, considering the presentation order.

**Condition/order**	**1** **Mean (SD)**	**2** **Mean (SD)**	**3** **Mean (SD)**	**4** **Mean (SD)**	**H** **-statistic**	**DF**	**p** ** value**⁣^∗^
Condition 1—SV0°	−2.4 (1, *n* = 16)	−1.9 (1.4, *n* = 16)	−1.5 (1.7, *n* = 20)	−2.2 (1.5, *n* = 14)	4.62	3	0.20
Condition 2—SV±90°	−11.7 (2.9, *n* = 13)	−12.3 (2.6, *n* = 20)	−10.8 (3, *n* = 16)	−12.8 (2.2, *n* = 17)	3.84	3	0.28
Condition 3—DV0°	−1.5 (1.5, *n* = 20)	−2.8 (1.5, *n* = 10)	−1.6 (1.2, *n* = 19)	−1.5 (1.5, *n* = 17)	6.43	3	0.09
Condition 4—DV±90°	−12 (2.5, *n* = 17)	−11.8 (2.7, *n* = 26)	−12.1 (2.2, *n* = 12)	−12.5 (1.8, *n* = 11)	0.37	3	0.95

Abbreviations: DF, degrees of freedom; SD, standard deviation.

⁣^∗^Kruskal–Wallis test.

**Table 7:** Descriptive measures (mean and standard deviation) for the SRT in the test and retest stages and the difference between them (test–retest) for the total sample (*N* = 22 children).

**PROSER**	**Test**		**Retest**		**Difference (retest-test)**		**p** ** value** ^**a**^
	**Mean**	**SD**	**Mean**	**SD**	**Mean**	**SD**	
Condition 1—SV0°	−2.16	1.70	−2.06	1.99	0.11	2.06	0.6023
Condition 2—SV±90°	−10.54	4.31	−11.38	4.29	−0.84	4.83	0.3003
Condition 3—DV0°	−1.48	1.60	−2.17	1.39	−0.68	1.43	0.0317
Condition 4—DV±90°	−10.65	3.47	−11.67	4.18	−1.03	4.47	0.1157
Spatial advantage	8.37	3.79	9.32	2.98	0.95	3.62	0.4077
Speaker advantage	−0.68	1.01	0.11	1.61	0.79	2.00	0.1074
Total advantage	8.49	3.14	9.62	3.04	1.13	3.51	0.1434

Abbreviation: SD, standard deviation.

^a^Wilcoxon's paired test.

**Table 8:** Mean and SD of the difference obtained in the test/retest for each condition and advantage of the PROSER test.

**PROSER**	**Difference (test–retest)**	
	**Mean**	**SD**
Condition 1—SV0°	0.11	2.06
Condition SV±90°	−0.84	4.83
Condition 3—DV0°	−0.68	1.43
Condition DV±90°	−1.03	4.47
Spatial advantage	0.95	3.62
Speaker advantage	0.79	2.00
Total advantage	1.13	3.51

Abbreviation: SD, standard deviation.

## Data Availability

The data used to support the findings of this study are available from the corresponding author upon request.
